# Treadmill Exercise Ameliorates Depression-Like Behavior in the Rats With Prenatal Dexamethasone Exposure: The Role of Hippocampal Mitochondria

**DOI:** 10.3389/fnins.2019.00264

**Published:** 2019-03-26

**Authors:** Tianwen Wu, Yan Huang, Yuxiang Gong, Yongjun Xu, Jianqiang Lu, Hui Sheng, Xin Ni

**Affiliations:** ^1^Department of Physiology, Second Military Medical University, Shanghai, China; ^2^School of Kinesiology, Shanghai University of Sport, Shanghai, China; ^3^Department of Clinical Genetics and Experimental Medicine, Fuzhou General Hospital, School of Medicine, Xiamen University, Fuzhou, China; ^4^Research Center of Molecular Metabolomics, Xiangya Hospital, Central South University, Changsha, China

**Keywords:** depression, hippocampus, glucocorticoid, mitochondria, exercise

## Abstract

Prenatal exposure to synthetic glucocorticoids (sGCs) can increase the risk of affective disorders, such as depression, in adulthood. Given that exercise training can ameliorate depression and improve mitochondrial function, we sought to investigate whether exercise can ameliorate depression-like behavior induced by prenatal sGC exposure and mitochondria function contributes to that behavior. At first, we confirmed that prenatal dexamethasone (Dex) administration in late pregnancy resulted in depression-like behavior and elevated level of circulatory corticosterone in adult offspring. We then found that mRNA and protein expression of a number of mitochondrial genes was changed in the hippocampus of Dex offspring. Mitochondria in the hippocampus showed abnormal morphology, oxidative stress and dysfunction in Dex offspring. Intracerebroventricular (ICV) injection of the mitochondrial superoxide scavenger mitoTEMPO significantly alleviated depression-like behavior but did not significantly affect circulatory corticosterone level in Dex offspring. The adult Dex offspring treated with treadmill exercise starting at four-weeks of age showed ameliorated depressive-like behavior, improved mitochondrial morphology and function and reduced circulatory corticosterone level. Our data suggest mitochondria dysfunction contributes to depression-like behavior caused by prenatal sGC exposure. Intervention with exercise training in early life can reverse depression caused by prenatal Dex exposure, which is associated with improvement of mitochondrial function in the hippocampus.

## Introduction

During late gestation, concentration of glucocorticoids (GCs) in the fetal circulation is exponentially increased, and this surge of GCs is critical for fetal organ development including the lungs and brain (Fowden and Forhead, 2009). Therefore, synthetic GCs (sGCs) are usually used to treat pregnant women at risk of preterm delivery in order to promote the development of fetal organs and impede preterm delivery associated morbid symptoms, such as respiratory distress syndrome and intra-ventricular hemorrhage (Amiya et al., 2016). However, excess GC exposure can disturb normal fetal neurodevelopmental progress and impact lifelong programming of neuroendocrine function and behavior (Asztalos, 2012; O’Donnell and Meaney, 2017). Emerging epidemiological evidence has indicated that prenatal exposure to increased amounts of sGCs causes increased susceptibility to neuropsychiatric disorders, such as anxiety and depression, in adulthood (Khalife et al., 2013; Braithwaite et al., 2017). Many animal studies have also demonstrated that repeated exposure to sGCs in late gestation leads to depression-like behavior and other modified behavior in adult offspring (Hiroi et al., 2016; Conti et al., 2017; Xu et al., 2018). Although a number of genes have been found to be associated with depression-like behavior (McCoy et al., 2017; Xu et al., 2018), the mechanisms underlying the prenatal GC programming of depression-like behavior remain largely unknown.

Mitochondria are integral eukaryotic organelles that play major roles in numerous cellular processes, particularly in aerobic energy production and thermogenesis (Hadj-Moussa et al., 2018). Of note, mitochondria are particularly important for the brain because of both its high levels of energy use and its inability to store large amounts of energy reserves in the form of glycogen. Moreover, mitochondria can generate reactive oxygen species (ROS) that may have toxic effects in cells (Allen et al., 2018). Mitochondria are involved in many processes including apoptosis and calcium homeostasis, which is necessary for the proper functioning of the nervous tissue (MacAskill et al., 2010; Xavier et al., 2016). A growing body of evidence has indicated that mitochondrial dysfunction would be a crucial factor in the development and progress of depression (Marazziti et al., 2011). It has been reported that patients with major depression disease (MDD) display changed mitochondrial morphology and function as well as have abnormal expression of genes encoding for mitochondrial proteins in the brain (Gardner and Boles, 2011). More recently, a number of studies have demonstrated that mitochondrial abnormalities may contribute to the depression-like behavior induced by chronic stress or prenatal stress (Gong et al., 2011; Chakravarty et al., 2013;Glombik et al., 2016).

So far, the therapeutic strategy for MDD is antidepressant therapy although it is unsatisfactory since most patients are partial responder to the treatment (MacQueen et al., 2017). Therefore, much effort in the past decades has focused on seeking adjuvant therapeutic approaches. The evidence unearthed so far suggests that exercise training is an adjuvant treatment approach for MDD as many clinical data have demonstrated that physical exercise as a regular life style lead to improvements in depressive status (Null et al., 2017). Animal studies have also shown that exercise training ameliorates depression-like behavior (Chen et al., 2016; Morgan et al., 2018). Similarly, using several rodent models of depression, we have demonstrated that exercise training reduces depressive behavior (Wang et al., 2016; Liu et al., 2018). Up to now, the biological mechanisms by which exercise ameliorates depression are yet unclear. However, Aguiar et al. (2014) demonstrated that exercise engages mitochondrial pathways, which might be associated to the anti-depressive effect of exercise.

The objectives of the present study were to explore the role of mitochondria in the development of depression-like behavior induced by prenatal exposure to sGCs and to evaluate the impact of exercise on the depression-like behavior programmed by sGCs. To achieve these goals, we set up a series of experiments to confirm that depression-like behavior can be induced by prenatal exposure to dexamethasone (Dex) in late pregnancy in rats, then clarified the contribution of mitochondria dysfunction to prenatal Dex programming depression-like behavior, and finally elucidated the association of mitochondria function with excise intervention of depression-like behavior caused by prenatal Dex exposure.

## Materials and Methods

### Animals

Adult Sprague Dawley rats weighing 220 ± 20 g were obtained from Shanghai Laboratory Animal (Shanghai, China). All animal procedures were carried out in accordance with the guidelines for the use of laboratory animals published by the People’s Republic of China Ministry of Health (January 25, 1998), with the approval of the Ethical Committee of Experimental Animals of the Second Military Medical University. Procedures were designed to minimize the number of animals used and their suffering. The rats were housed in social groups of 3 to 5 in a cage with regular light–dark cycles (lights on at 7:00 AM, lights off at 7:00 PM) under controlled temperature (22 ± 2°C) and humidity (50 ± 10%), and were provided standard diet and water *ad libitum*. Breeding females were handled daily for 1 week. The female was placed with a male at 3:00 PM. The male was removed the next day at 8:00 AM to its social group, and the female was transferred to a new cage. Pregnant rats were confirmed by microscopic analysis of vaginal smears for the presence of sperm, then were assigned randomly to control (Con) and dexamethasone (Dex) groups. Dexamethasone-21-phosphate disodium salt (Sigma-Aldrich, St. Louis, MO, United States) was dissolved in saline to achieve the concentration as 100 μl containing 0.13 mg/kg body weight. The dosage of Dex was chosen on the basis of the literature (Hauser et al., 2006; Liu et al., 2018) and our preliminary study. During gestational days (GD) 14 to 21, rats of DEX group were subcutaneously administered with 0.13 mg/kg dexamethasone-21-phosphate disodium salt (equal to 0.1 mg/kg dexamethasone), while control rats were received vehicle (0.9% saline) once a day for 7 days. The pregnant rats delivered undisturbed to produce the offspring. Given that estrus cycle may have impact on the behavior tested and the expression of various factors, the male offspring were used in the following experiments.

### Exercise Protocol

Twenty male offspring rats at 4 weeks of age were randomly selected from Dex group. They were then randomly assigned to two groups (*n* = 10 for each group): sedentary and exercise. The exercise protocol for moderate physical activity was performed as described by prior studies (Ji et al., 2017; Shin et al., 2017). Briefly, animals were put on the treadmill at a speed of 2 m/min for the first 5 min, flow up a speed of 5 m/min for the next 5 min, and a speed of 8 m/min for the last 20 min, with the 0° inclination. The animals were adapted to the treadmill for a week, and then the above exercise was performed for 5 days a week for four weeks. After exercise, behavioral tests were examined on all of the animals. Then, rats were decapitated at 12:00 PM to 13:00 PM on the day of sacrifice for collection of blood and tissue. Hippocampi were rapidly and carefully separated on an ice-plate and then stored at -80°C until assays. The blood was collected and the serum was separated and stored at -80°C until assays.

### Behavioral Tests

The behavior of offspring was monitored at 9 weeks. The forced swimming test (FST) and sucrose preference test were performed as previously described (Drake et al., 2005; Long et al., 2013). In the FST, each rat was introduced for 5 min into separate transparent cylindrical containers (diameter 30 cm, height 45 cm) that were filled with water to 30 cm so that rats could only touch the bottom with the tip of the tail. Water temperature was maintained at 24 ± 2°C. Duration of immobility was measured. The sucrose preference test was used to test behavioral anhedonia. Briefly, 2 bottles of 1% sucrose solution were placed in each cage, and 24 h later, one bottle was replaced with tap water for 24 h. After adaptation, rats were deprived of water and food for 24 h, followed by the sucrose preference test, in which rats housed in individual cages had free access to 2 bottles, one containing 200 ml of sucrose solution (1% w/v) and the other 200 ml of water. At the end of 24 h, sucrose and water consumption was measured, and the sucrose preference was calculated as the volume of 1% sucrose solution consumed expressed as a percentage of the total liquid intake.

### Hormone Assays

Corticosterone levels in serum were measured using a commercially available radioimmunoassay kit (Sino-United Kingdom Institute of Biologic Technology, Beijing, China) according to the manufacturer’s instructions. The specificity of the kit was 100% for corticosterone, and it did not cross react with progesterone, aldosterone, cortisol, testosterone, cortisone, dehydroepiandrosterone (DHEA), DHEA-sulfate, or pregnenolone. Intraassay variation was <7.5% and interassay variation <9.5%.

### Microarray Analysis

The transcriptional profiles of hippocampal tissues from the male Dex offspring were characterized by Affymetrix v2 U133 plus 2 gene arrays using the One-Cycle Eukaryotic Target Labeling Assay protocol (Affymetrix, Santa Clara, CA, United States).

### Total RNA Extraction and Real-Time Quantitative PCR

Total RNA of hippocampus of male offspring was extracted using Trizol reagent (Thermo Fisher Scientific, Waltham, MA, United States) following the manufacturer’s instructions. Genomic contamination was removed by column treatment of RNA samples with DNase for 20 min at 20°C, using the DNAeasy cleanup protocol following the manufacturer’s instructions (Qiagen, Germantown, MD, United States). RNA purity and concentrations were assessed using spectrophotometric analysis, and integrity was verified using gel electrophoresis. Briefly, 2 μg RNA was reverse transcribed with oligo(dT)12-18 primer using the Moloney murine leukemia virus reverse transcriptase (Promega, Madison, WI, United States). Real-time PCR was performed using SYBR green (Hoffmann-La Roche, Basel, Switzerland) incorporation with a CFX96 real-time detection system (Bio-Rad, Hercules, CA, United States). The reaction solution consisted of 2.0 μl diluted cDNA product, 0.1 μM of each paired primer, 100 μM deoxynucleotide triphosphates, 1 U Taq DNA polymerase (Promega), and 10.0 μl PCR buffer. The primer sequences for uncoupling protein 3 (UCP3), isocitrate dehydrogenase 2 (IDH2) and solute carrier family 25 member 21(Slc25a21), were designed based on cDNA sequences in GeneBank. The following primers were used: UCP3 (accession number NM_013167): sense 5′- CAAAGGAACGGACCACTCCA-3′ and anti-sense 5′- CTCCAGTTCCCAGGCGTATC-3′, IDH2 (accession number NM_001014161.1): sense 5′- AAGCCCATCACCATTGGCAG-3′ and anti-sense 5′- CCGAAATGGACTCGTCGGTG-3′, Slc25a21(accession number NM_133614): sense5′- AGGAAGACTTAGGTATGAGTCAGAA-3′ and anti-sense 5′- TCACATTTTAACTCAGCACTCACAA-3′, β-actin (accession number NM_001101): sense 5′- TGTGTTGGCGTACAGGTCTTTG-3′ and anti-sense 5′- GGGAAATCGTGCGTGACATTAAG-3′. The temperature range to detect the melting temperature of the PCR product was set from 60 to 95°C. The housekeeping gene β-actin was measured for each sample as an internal PCR control for sample loading and normalization. The specificity of the primers was verified by examining the melting curve as well as by subsequent sequencing of the PCR products. To determine the relative quantitation of genes expression for both target and housekeeping gene, the comparative C_t_ method with arithmetic formulae was used (Livak and Schmittgen, 2001). Subtracting the C_t_ of the housekeeping gene from the C_t_ of the target gene yields the ΔC_t_ in each group, which was entered into the equation 2^-ΔΔCt^ and calculated for the exponential amplification of PCR. β-Actin was used for calculation of ΔC_t_ in presentation of results.

### Western Blotting Analysis

Hippocampal tissues of male offspring were homogenized in the presence of lysis buffer consisting of 60 mM Tris–HCl, 2% sodium dodecyl sulfate (SDS), 10% sucrose, 2 mM phenylmethylsulfonyl fluoride (Merck, Darmstadt, Germany), 1 mM sodium orthovanadate (Sigma-Aldrich) and 10 μg/ml aprotinin (Bayer, Leverkusen, Germany). The lysates were quickly centrifuged at 4°C. The supernatant was collected and protein concentration was assayed using a modified Bradford assay. The samples were then diluted in sample buffer (250 mM Tris–HCl, 4% SDS, 10% glycerol, 2% β-mercaptoethanol and 0.002% bromophenol blue) and boiled for 10 min. Protein load was 30 μg per lane in 10% SDS-PAGE and subsequently transferred to nitrocellulose membranes. The blots were blocked with 5% skim milk powder in 0.1% Tris-buffered saline/Tween 20 at room temperature for 2 h, then incubated with antibodies against UCP3 (ab3477, Abcam), IDH2 (ab131263, Abcam) and Slc25a21(ab167033, Abcam) at a dilution 1:500 to 1:1000 overnight at 4°C. After 3 washes with Tris-buffered saline/Tween 20, the membrane was incubated with the secondary antibodies of horseradish peroxidase–conjugated antibody for 1 h at room temperature. Immunoreactive proteins were detected and visualized using the enhanced chemiluminescence western blot detection system (Santa Cruz Biotechnology). To control sampling errors, the ratio of band intensities to β-tubulin or GAPDH (Sigma-Aldrich) was obtained to quantify the relative protein expression level.

### Isolation of Mitochondria

Mitochondria of hippocampus were isolated using Mitochondria Fractionation Kit (Beyotime) as previously described (Du et al., 2016). Briefly, the hippocampus tissues were quickly removed from adult male offspring and placed in chilled isolation media (0.25 M sucrose, 10 mM Tris–HCl buffer, pH 7.4, 1 m MEDTA, and 250 μg BSA/ml). The tissues were minced and washed with the isolation medium, and 10% (w/v) homogenates were ready. Nuclei and cell debris were sedimented by centrifugation at 600 *g* for 10 min at 4°C and discarded. The supernatant was subjected to centrifugation at 10,000 *g* for 10 min at 4°C. The resulting mitochondrial pellets were suspended in the isolation medium.

### Detection of Mitochondrial Superoxide Production

MitoSOX (Molecular Probes, Invitrogen, Carlsbad, CA, United States), a cell-permeable probe, can accumulate specifically in mitochondria and become fluorescent after oxidation by superoxide. MitoSOX was dissolved in DMSO immediately before use, and then added into isolated mitochondria at a final concentration of 5 μM (DMSO diluted to less than 0.1%). After 30 min, the media were replaced with 100 μl HEPES buffered saline (10 mM HEPES, pH 7.4, 150 mM NaCl, 5 mM KCl, 1 mM MgCl_2_, and 1.8 mM CaCl_2_); then, red fluorescence was obtained at 485 nm excitation and 590 nm emission using a Synergy TM fluorescence plate reader (Bio-Tek Instruments).

### Assessment of Mitochondrial Membrane Potential

Mitochondrial membrane potential was detected with the fluorescent probe JC-1 (Sigma-Aldrich), which exists predominantly in monomeric form in cells with depolarized mitochondria and displays fluorescent green. Cells with polarized mitochondria predominantly contain JC-1 in aggregate form and show fluoresced red. Loading was done by incubating isolated mitochondria with 2 μM JC-1 for 15 min. After staining, the red fluorescent signals were excited at 530 nm and detected at 630 nm, and the green fluorescence was excited at 488 nm and detected at 530 nm using a Synergy TM fluorescence plate reader. The ratio of red and green fluorescence indicates mitochondrial membrane depolarization.

### Measurement of Mitochondrial ATP Concentration

Isolated mitochondria were homogenized in a protein extraction solution (Pierce). The supernatant after centrifugation at 10,000 *g* for 10 min was subject to determination of ATP concentration, using an ATP bioluminescence assay (Beyotime). Light emitted from a luciferase-mediated reaction was measured by a tube luminometer (Tecan).

### Intracerebroventricular (ICV) Injection

Eighteen male offspring were randomly picked up from Dex group and then randomly divided into 3 groups (*n* = 6 in each group): vehicle and mitoTEMPO (0.02 and 0.2 μmol/kg). They were anesthetized with 10% chloral hydrate (0.33 mg/kg, i.p.) and placed in a stereotaxic apparatus (Stoelting Co., Wood Dale, IL, United States). At first, stainless steel guide cannulas were implanted in lateral ventricle using stereotactic co-ordinates (AP = –0.8 mm, L = +1.5 mm, and DV = –3.6 mm) (Stengel et al., 2011), and fixed to the skull with dental cement and one metal screw. After one week recovery, the rats were anesthetized continuously with 2.5% halothane, and then mitoTEMPO (Sigma-Aldrich) at the dosage 0.02 or 0.2 μmol/kg (5 μL) was injected into the left ventricle at the rate of 1 μL/min by Hamilton syringe. Equal volume of saline was injected into the left ventricle of the vehicle animals. The infusion needle was left in place for an additional 5 min after each infusion and then slowly withdrawn. This injection was taken once a day for 4 days. Then, behavioral tests were performed on all of animals.

### Electron Microscopy

Hippocampus specimens were immediately placed in 2.5% glutaraldehyde in 0.1 M cacodylate buffer, sectioned to ∼1 mm^2^, and incubated in the same glutaraldehyde solution for 12 h at room temperature. Samples were postfixed in 1% osmium tetroxide for 1.5 h, then dehydrated in increasing concentrations of alcohol, immersed in propylene oxide, and embedded in Araldite 502 resin at 60°C. Ultrathin sections were placed on grids and stained with uranyl acetate and lead citrate. The ultrastructure of hippocampus was observed under a transmission electron microscope (JEOL 1010; JEOL, Akishima, Japan).

### Statistics

Data were presented as mean ± SEM. All data were tested for homogeneity of variance by the Bartlett test at first, and then analyzed using one or two-way ANOVA followed by Dunnett’s or LSD *post hoc* test, where appropriate. *P* < 0.05 was considered significant.

## Results

### Prenatal Dex Exposure Induces Depression-Like Behavior and Increases Circulatory Corticosterone Level

**Figure 1:**
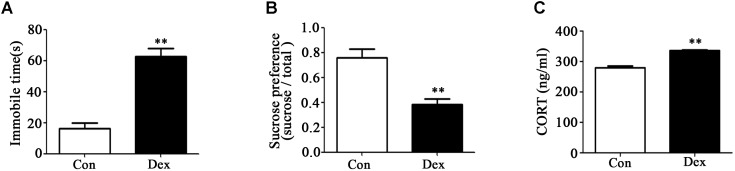
Prenatal Dex exposure induces depression-like behavior and increases circulatory GC level in adult offspring. Pregnant rats were administered Dex at 0.1 mg/kg/d during GD14 to 21. Behavioral tests and determination of corticosterone concentration in blood were performed on male offspring rats at adult (9-week old). Immobile time of FST **(A)**; sucrose preference test **(B)**; corticosterone level **(C)**. Data are presented as means ± SEM (*n* = 10). ^∗∗^*P* < 0.01 vs. control. Con, control; Dex, dexamethasone.

As shown in Figure 1A,B, immobile time in the FST was significantly increased whilst sucrose preference was significantly reduced in male Dex offspring compared with control offspring (*P* < 0.01).

It has been reported that increased hypothalamic–pituitary–adrenal (HPA) activity contributes to depression-like behavior induced by prenatal GC exposure (Shoener et al., 2006; Xu et al., 2018). We therefore examined circulating corticosterone level in offspring with prenatal Dex exposure. As shown in Figure 1C, corticosterone level in circulation was significantly higher in Dex offspring than that in control offspring (*P* < 0.01).

### Prenatal Dex Exposure Leads to Mitochondrial Dysfunction in Hippocampus

**Figure 2:**
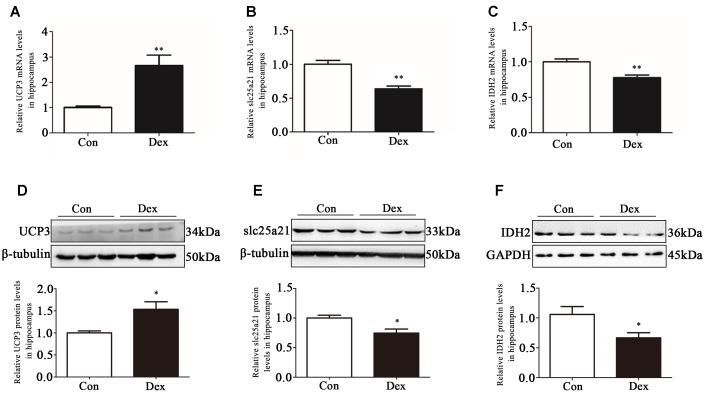
Effects of prenatal Dex exposure on hippocampal UCP3, Slc25a21 and IDH2 expression in adult offspring. Pregnant rats at GD 14 to 21 were subcutaneously administered either 0.1 mg/kg/d Dex or vehicle (0.9% saline) once a day for 7 days. Hippocampal tissues were obtained from adult offspring rats (9-week old). UCP3 **(A)**, Slc25a21 **(B)**, and IDH2 **(C)** mRNA level was determined by Q-PCR. Protein level of UCP3 **(D)**, Slc25a21 **(E)**, and IDH2 **(F)** was determined by western blot analysis. Representative protein bands are presented above corresponding histogram. Data are presented as means ± SEM (*n* = 10/group). ^∗^*P* < 0.05, ^∗∗^*P* < 0.01 vs. control. Con, control; Dex, dexamethasone.

The hippocampus is the key brain region responsible for affective and behavioral functions and is sensitive to steroid hormones (Miller and O’Callaghan, 2005; Snyder et al., 2011). To identify the key genes in hippocampus that mediate depression-like behavior, we performed microarray analysis to compare the gene expression profiles in hippocampus of Dex offspring and control offspring (Xu et al., 2018). Among the regulated genes, UCP3, Slc25a21 and IDH2 are the genes related to mitochondrial function. Using Q-PCR, we confirmed that UCP3 expression was significantly upregulated whilst Slc25a21 and IDH2 expression was significantly downregulated in Dex offspring compared with control offspring (*P* < 0.01, Figure 2A–C). Consistently, western blotting analysis showed that the protein level of UCP3 was significantly increased whilst the levels Slc25a21 and IDH2 were significantly decreased in Dex offspring compared with control offspring (*P* < 0.05, Figure 2D–F).

**Figure 3:**
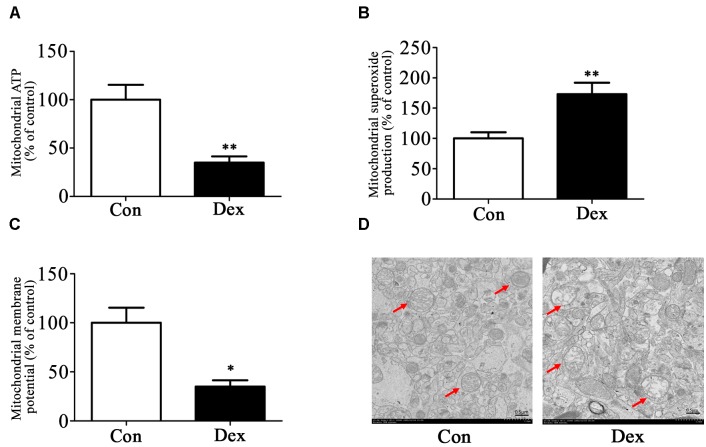
Effects of prenatal Dex exposure on mitochondrial function and morphology in hippocampus of adult offspring. Pregnant rats at GD 14 to 21 were subcutaneously administered either 0.1 mg/kg/d Dex or vehicle (0.9% saline) once a day for 7 days. Mitochondria were isolated from the hippocampus of adult offspring rats (9-week old) for determination of ATP production **(A)**, mitochondrial superoxide production **(B)**, and membrane potential **(C)** as described in Section “Materials and Methods”. Mitochondrial morphology **(D)** was determined by transmission electron microscopy. Data are presented as means ± SEM (*n* = 10/group). ^∗^*P* < 0.05, ^∗∗^*P* < 0.01 vs. control. Con, control; Dex, dexamethasone.

Next, we assessed the effect of prenatal Dex exposure on mitochondrial function in the hippocampus. As shown in Figure 3A–C, prenatal Dex exposure resulted in a significant decrease in ATP production (*P* < 0.01 vs. control) and mitochondrial membrane potential (*P* < 0.05 vs. control), as well as a significant increase in mitochondrial superoxide production (*P* < 0.01 vs. control).

The ultrastructure of mitochondria in hippocampus was then determined by transmission electron microscopy. As shown in Figure 3D, male Dex offspring showed mitochondrial damage, including a decrease in cristae density or even disappearance, vacuole formation by mitochondrial outer membrane extension, and intermembrane space expansion (Figure 3D).

### MitoTEMPO Administration Ameliorates Depression-Like Behavior in Dex Offspring

**Figure 4:**
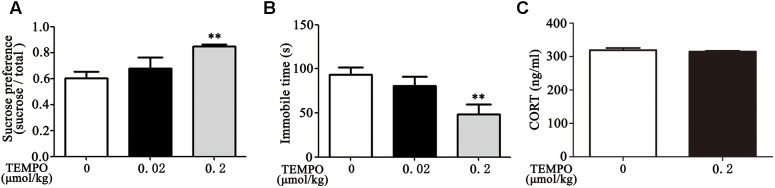
ICV administration of MitoTEMPO improves depression-like behavior and reduces circulatory corticosterone level in Dex offspring. Male Dex offspring received ICV administration of MitoTEMPO (0.02 or 0.2 μmol/kg, 5 μL/day) for 4 days. Sucrose preference test **(A)**, FST **(B)** and corticosterone concentration in blood **(C)** were then determined. Data are presented as means ± SEM (*n* = 6/group). ^∗∗^*P* < 0.01 vs. control.

MitoTEMPO is a mitochondria-targeted antioxidant with superoxide and alkyl radical scavenging properties (Hu and Li, 2016). We then examined the effect of MitoTEMPO administration on depression-like behavior in Dex offspring. Adult Dex rats were received ICV injection of MitoTEMPO (0.02 or 0.2 μmol/kg, 5 μL) for 4 days prior to behavioral tests. As shown in Figure 4A,B, injection of MitoTEMPO at the dosage of 0.02 μmol/kg had no significant effect on depression-like behavior, while administration of 0.2 μmol/kg MitoTEMPO significantly decreased depression-like behavior as evidenced by increased sucrose preference and a decrease in immobile time in FST compared to those with vehicle injection.

Given that increased corticosterone level in circulation contributes to depression-like behavior, we examined the level of corticosterone in response to MitoTEMPO administration. As shown in Figure 4C, there was no significant difference in corticosterone level among injection with 0.2 μmol/kg MitoTEMPO and vehicle injection groups.

### Treadmill Exercise Ameliorates Depression-Like Behavior, Reduces Circulatory Level of Corticosterone and Improves Mitochondrial Function in Dex Offspring

**Figure 5:**
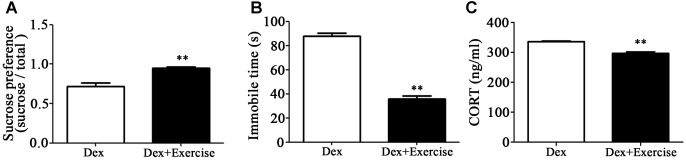
Effects of treadmill exercise on depression-like behaviors and circulatory corticosterone level in Dex offspring. Dex offspring at 4-week old received 4 weeks of treadmill exercise. Then, sucrose preference test **(A)** and FST **(B)**, and corticosterone concentration in blood **(C)** were determined. Data are presented as means ± SEM (*n* = 6/group). ^∗∗^*P* < 0.01 vs. Dex. Dex, dexamethasone; CORT, corticosterone.

As mentioned, exercise could be an adjuvant treatment approach for depression, we therefore applied treadmill exercise to 4-week old Dex offspring in order to determine the impact of exercise on depression-like behavior in early life. As shown in Figure 5A,B, these rats with exercise training displayed improvement of depression-like behavior in adult. Sucrose preference was significantly increased and the immobile time in FST was significantly decreased in Dex offspring with exercise training compared those in Dex offspring without exercise training. In addition, circulatory level of corticosterone was lower in Dex offspring with exercise than that in Dex offspring without exercise (Figure 5C).

**Figure 6:**
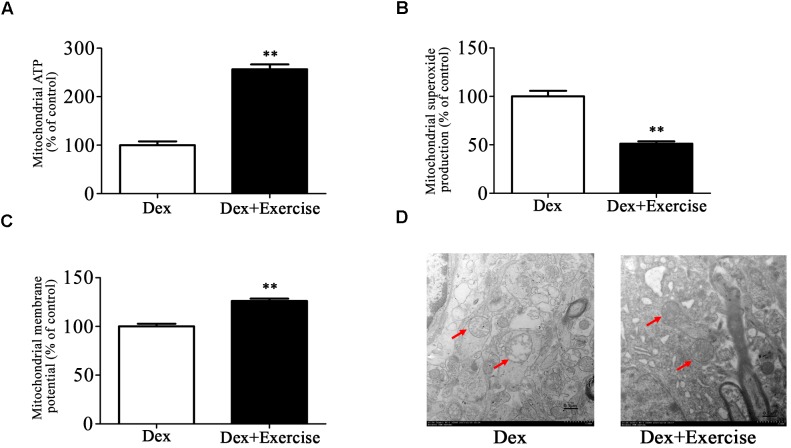
Effects of treadmill exercise on mitochondrial function and morphology in Dex offspring. Dex offspring at 4-week old received 4 weeks of treadmill exercise. Then, mitochondria were isolated from hippocampus for the determination of ATP production **(A)**, mitochondrial superoxide production **(B)**, and membrane potential **(C)** as described in Section “Materials and Methods”. Mitochondrial morphology **(D)** was determined by transmission electron microscopy. Data are presented as means ± SEM (*n* = 10/group). ^∗∗^*P* < 0.01 vs. Dex. Dex, dexamethasone.

We then assessed the effect of treadmill exercise on mitochondrial function and morphology in hippocampus. As shown in Figure 6A–C, Dex offspring with treadmill exercise showed a significant increase in ATP production and mitochondrial membrane potential and a significant decrease in mitochondrial superoxide production compared with those without exercise (*P <* 0.01).

Electron microscopy analysis showed that mitochondrial morphology in hippocampus was greatly improved in Dex offspring with exercise compared with those without exercise (Figure 6D).

## Discussion

In the present study, we confirmed that prenatal GCs exposure induces depression-like behavior and increases HPA activity, demonstrated that mitochondrial dysfunction in hippocampus occurred in Dex offspring, and found that depression-like behavior was ameliorated by administration of a mitochondria-targeted antioxidant into the brain. Moreover, we also showed that treadmill exercise intervention in early life can reverse the above behavior outcome and mitochondrial alternations in brain. Our data indicates that mitochondria dysfunction contributes to Dex programming behavior and that exercise reversion of this behavior outcome is associated with recovery of mitochondrial function and morphology in the hippocampus.

As mentioned previously, the hippocampus is the key brain region in cognitive, affective and behavioral functions and is a highly plastic structure that is sensitive to steroid hormones (Miller and O’Callaghan, 2005; Snyder et al., 2011). Our previous study has shown that prenatal Dex exposure leads to significant changes in 149 genes in hippocampus (Xu et al., 2018). We noted that several genes including UCP3, IDH2 and Slc25a21 are the regulators of mitochondrial function, glycolysis and oxidative stress. UCP3 belongs to a family of mitochondrial transporter proteins that mediates a regulated permeabilization of the mitochondrial inner membrane to protons (Chakravarty et al., 2013; Giralt and Villarroya, 2017). IDH2 is an NADP(+)-dependent mitochondrial protein that regulates mitochondrial redox status through its role in intermediary metabolism and energy production (Han et al., 2017). Slc25a21 is a member of the mitochondrial carrier subfamily of solute carrier protein genes and functions as a gated pore, translocating ADP from the mitochondrial matrix into the cytoplasm to maintain the cytoplasmic phosphorylation potential for cell growth (Gutierrez-Aguilar and Baines, 2013). Consistent with the functional role of these factors, we found that hippocampal mitochondria exhibited oxidative stress and impairment of energy production as evidenced by an increase in ROS production and a decrease in ATP production and reduced mitochondrial membrane potential in Dex offspring. It is known that increased ROS content would cause mitochondria damage such as leading to swollen and reduction of cristae density (Wang et al., 2012). Consistently, morphology of mitochondria showed a dramatic reduction of cristae density and vacuole formation by mitochondrial outer membrane extension and intermembrane space expansion in Dex offspring. As UCP3 plays an important role in permeabilization of the mitochondrial inner membrane to protons, the above morphological changes are at least partly attributed to changed UCP3 expression in Dex offspring.

Mitochondria are essential for the life of the cell as they are well-known ATP producers. In the brain, mitochondria are also crucial for the processes of neuroplasticity, including neural differentiation, neurite outgrowth, neurotransmitter release and dendritic remodeling (Mattson et al., 2008). Given that depression development is associated with abnormal neurogenesis and synaptic formation in some brain regions (Pilar-Cuellar et al., 2014; Gulbins et al., 2015), increasing lines of evidence have indicated that mitochondrial disturbances are involved in the development and progress of depression (Gardner and Boles, 2011; Marazziti et al., 2011). In animal models of depression induced by chronic mild stress, the collapse of mitochondrial membrane potential, inhibited mitochondrial respiration rates and destroyed mitochondrial ultrastructure have been reported (Gong et al., 2011). In the model of LPS-induced depression, mitochondrial dysfunction is believed to play a central role in the pathogenesis of depressive behavior (Chen et al., 2017). Some studies have also shown that prenatal stress resulted in mitochondrial malfunction, which may play a role in depression (Chakravarty et al., 2013; Glombik et al., 2016). In the present study, we have also provided the evidence that mitochondria dysfunction contributes to depression-like behavior caused by prenatal Dex exposure.

There is now substantial evidence that exercise could effectively ameliorate depressive symptoms (Chen et al., 2016; Null et al., 2017; Morgan et al., 2018). Our previous studies also showed that swimming exercise is beneficial to depression-like behavior induced by prenatal Dex exposure (Liu et al., 2013). The present study showed that treadmill exercise can also improve depression-like behavior induced by prenatal Dex exposure. Studies from Wen et al. (2014) have indicated that anti-depressive actions of exercise may, in part, be due to a reversal of mitochondrial dysfunction. Moreover, a number of studies have shown that exercise could improve mitochondrial function and increased anti-oxidant enzymes in the nervous tissue. For instance, Dos Santos et al. (2017) showed physical exercise during the developmental period may protect against brain oxidative damage caused by chronic stress exposure later in life. In the present study, we showed that exercise ameliorates depression-like behavior whilst improves mitochondrial function in Dex offspring, indicating that improvement of mitochondrial function contributes to exercise ameliorating the behavior programmed by prenatal sGC exposure.

Many studies have demonstrated that prenatal sGC exposure programs the HPA axis and subsequently leads to increased HPA activity (Liu et al., 2013; van Bodegom et al., 2017). It is known that increased HPA activity is one of key factors responsible for development of depression (Keller et al., 2017; Juruena et al., 2018). Here, we showed that exercise also reduced circulatory level of corticosterone in Dex offspring, which suggests that exercise improvement of depression-like behavior is also associated with reversion of higher HPA activity in Dex offspring. Of note, we found that administration of the mitochondria-targeted antioxidant MitoTEMPO could not reduce circulatory level of corticosterone in Dex offspring, suggesting that mitochondria dysfunction may not be involved in increased HPA activity caused by prenatal Dex exposure. The mechanisms underlying prenatal sGC programming HPA activity remain to be further investigated.

In conclusion, we found that prenatal sGC exposure results in depression-like behavior, accompanied by alterations in mitochondrial function, and that intervention with exercise improves both depression-like behavior and mitochondrial dysfunction. Our results suggest that exercise may recover mitochondrial function, thereby ameliorating depression caused by prenatal sGC exposure.

## Data Availability

Publicly available datasets were analyzed in this study. This data can be found here: https://doi.org/10.1096/fj.201700948RR.

## Author Contributions

TW, YH, and YG performed the experiments and analyses. TW wrote the manuscript. XN and HS designed the experiments and made the final revision. YX and JL participated in discussions and revisions of the article.

## Conflict of Interest Statement

The authors declare that the research was conducted in the absence of any commercial or financial relationships that could be construed as a potential conflict of interest.
